# A global network of scholars? The geographical concentration of institutes in migration studies and its implications

**DOI:** 10.1186/s40878-023-00336-1

**Published:** 2023-06-01

**Authors:** Lorenzo Piccoli, Didier Ruedin, Andrew Geddes

**Affiliations:** 1 Migration Policy Centre at the European University Institute, Via delle Fontanelle 19, 50014 San Domenico di Fiesole, Italy; 2 University of Neuchâtel, Rue Abram-Louis-Breguet 2, 2000 Neuchâtel, Switzerland

**Keywords:** Migration, Migration studies, Knowledge production, Mapping, Research

## Abstract

The study of international migration and responses to it has experienced rapid growth in the last three decades: an institutionalisation of migration studies. This paper identifies and specifies *infrastructural* and *semantic* elements of institutionalisation by establishing a global Directory of Migration Research Institutions identifying 282 institutes focused on migration research that were operative between 1945 and 2020. We observe a clear geographical concentration in the Americas and Europe and find that most institutes are in countries with higher economic development (GDP) and net immigration (not emigration). Using this evidence, we suggest that the institutionalisation of migration studies is driven by concerns and ideas produced in high-income ‘destination’ countries. We thus show that uneven knowledge production in migration studies is not only caused by exclusive categories, language, or journal policies, but also by a structural problem at an earlier stage: because of fewer resources invested in the creation of institutionalised academic knowledge structures, lower income countries have fewer possibilities to shape the semantic features of the field of migration studies, by which we mean the identification of subjects of interest, concepts, narratives, and priorities.

## Introduction

The study of international migration in its various forms has experienced a rapid increase in activity and centrality, particularly within the humanities and social sciences. Over the last three decades in particular, international migration has become the main object of research for hundreds of specialised centres and institutes. Scholars have already linked the outputs of these centres and institutes to the institutionalisation of ‘migration studies’ as a multidisciplinary field of study or as part of the ‘coming of age’ of the field of migration studies (King, 2015; Levy et al., 2020; Pisarevskaya et al., 2019; Vertovec, 2020).

In this article we further develop these perspectives by asking two, inter-related questions. First, what are the key dimensions of the institutionalisation of the field of ‘migration studies’? And, second, what are the consequences for knowledge production in the field? We provide a comprehensive picture of *where* migration knowledge is produced but also the crucial and related issue of *what kinds* of knowledge are produced. By this we mean the primary foci of research, which, as we show, tend to favour the perspective of higher income ‘destination’ countries on issues around the regulation of migration and associated rights frameworks. We thus specify and elaborate the meaning of institutionalisation: an *infrastructural* element linked to the creation of institutes dedicated to teaching, training and/or research on international migration; and, second, a *semantic* institutionalisation of migration studies that structures the intellectual organisation of the field, by which we mean the identification of subjects of interest, concepts, narratives, priorities and, equally importantly, the potentially distorted perspective from which these are viewed.

To address these questions, we created an original global Directory of Migration Research Institutions (DMRI) covering the period 1945–2020 (Piccoli, 2023). The DMRI identifies 282 research institutes with reference to international migration in their title and activities that serve as venues for the training of researchers and for research on migration. The DMRI dataset comprises information on the location, year of establishment, the size in terms of number of staff, the core themes of research and training.

We recognise that research on international migration occurs in other contexts, such as through the activities of individual scholars outside formally constituted centres or institutes; but, as this article makes clear, dedicated institutes are key nodes for the organisation of ‘migration studies’ and the semantic composition of the field with important consequences for the perspectives on international migration and the definition of challenges, subjects of interest, issues and themes. In addition, these centres and institutes often engage in teaching and training of the next generation of researchers and experts. Thus, to some extent they act as an entry level and gatekeepers by selecting aspiring experts and, of course, introducing them to a field of semantically institutionalised research. Despite their central role in the process of knowledge production, no study has systematically analysed the geographical distribution of centres and institutes dedicated to the study of international migration.

The article seeks to contribute to what has been referred to as a reflexive turn in migration studies and to the work around the specific epistemological and political ideas behind the growth of migration studies. We reveal the scale, size, scope, composition and geographical patterns of dedicated research centres and institutes. By doing so, we show how the infrastructural and semantic institutionalisation of migration studies: (1) consolidates specific concepts, questions, and topics through adaptation to practices perceived to have been successful elsewhere; and, (2) creates and reproduces geographical boundaries that delimit the circulation of ideas and resources.

## Knowledge production in migration studies

A rapid expansion of research on migration has been seen as evidence of the institutional development or ‘coming of age’ of the field of migration studies (King, 2015; Levy et al., 2020; Pisarevskaya et al., 2019; Vertovec, 2020). This process encompasses a range of activities and infrastructures, including conferences, journals, institutes, and study programmes. Over time, scholars have observed a clear growth in the volume of such activities (Pisarevskaya et al., 2019) and in self-referentiality within the field (Levy et al., 2020).

This process is not without tension: The institutionalisation of knowledge may reproduce hegemonic power relations, structural inequalities, and mechanisms of political exclusion, or it may critically challenge them. It has been observed, for example, that ‘even if the majority of the world’s refugees and migrants and the bulk of humanitarian interventions are located in the south, southern-based scholars are hard to find in the leading (i.e., most broadly cited) scholarly journals on the topic’ (Landau, 2019, p. 26; see also: Fiddian-Qasmiyeh, 2020; Shivakoti & Milner, 2021). Existing studies demonstrate that scholars in the Global South have a problem of access to knowledge in terms of bibliographies (Levy, 2020; McNally & Rahim, 2020; Pisarevskaya et al., 2019; Schmiz et al., 2020), journal submissions (Vargas-Silva, 2019), scholarly networks (Levy et al., 2020; Vargas-Silva, 2019), foundational concepts and ideas (Dahinden et al., 2021; Schinkel, 2018), maps (van Houtum & Bueno Lacy, 2020), and data collection exercises (Scheel, 2019). These studies reveal that the institutionalisation of migration studies is, in many ways, skewed, uneven, or, as Kofman (2020) puts it, ‘unequal’—of the kind that can be traced back to colonial knowledge production (Smith, 2021; Collins, 2022).

Despite the attention dedicated to this process of institutionalisation and its infrastructural and semantic effects, we still lack a comprehensive mapping of dedicated centres and institutes that pool resources for training researchers with special focuses on migration (Chan, 2020). Such institutes play a key part in the field of migration studies for two reasons. First, international migration is a complex phenomenon, characterised by uncertainty and loaded with moral and normative questions. In this context, the role of ‘experts’ can be important for policy-makers attempting to weigh alternative scenarios, although it is unclear to which extent scientific knowledge actually informs policy (Boswell et al., 2011). What is clear, however, is that research on various aspects of international migration has often been driven by the needs of governments and administrations, which becomes manifest in the objective of ‘policy-relevance’ and funding streams dedicated to research that fits with these policy concerns. There is also a troubled relationship between scientific research and policy with frequent calls for evidence-based policy, but also frequent laments from academics that policy does not pay attention to research findings.^1^ It can be said, however, that dedicated centres and institutes actively participate in the production of concepts, theoretical innovations, development of new methods and compilation of data sources that have important implications for policy and practice. This does not mean that centres and institutes all behave in the same way, or that all researchers seek to engage with policy (this is clearly not the case), but we argue that the availability of funding can and does play an important role in defining research priorities.

At the same time, and necessarily also influenced by the availability of funding and the themes identified by funders as being of importance, centres and institutes have a generative role: Through a set of formal and informal exchanges, they form and select—or contribute to the selection of—aspiring researchers. For these reasons, mapping the institutes that are dedicated to conducting research or providing training on international migration in its various forms can contribute to understanding more clearly the dynamics of institutionalisation of migration studies and the related processes of inclusion and exclusion. As we argue, these inclusions and exclusions are multi-faceted and can intersect with gendered and racialised inclusions, exclusions and omissions (Anthias, 2012; Cundill et al., 2021).

As we have already noted, institutionalisation possesses two linked dimensions: First, the infrastructural dimension has already been captured by work on the institutionalisation of migration studies that explores the increased number of research outputs as well as the growth in venues for the presentation and discussion of those outputs at various conferences, workshops and seminars. We extend this infrastructural dimension to consider the extent of institutionalisation that is evident through the creation of research centres and institutes with a focus on international migration or specific aspects of it and that can also combine their research role with research and training. There is, however, a second important dimension that we refer to as semantic: the ways in which the infrastructural dimension of institutionalisation shapes the conceptual constitution of the migration studies field, and thus the underlying meanings that are associated with it. These meanings have important implications for perspective and participation: from where are ‘issues’ viewed and by whom?

This is not to say that all centres and their members share a unified perspective. In fact, even the most cursory overview of the work of the centres that we identify demonstrates the diversity of approaches within them. Our point is that the location and broad thematic focus of research centres and institutes can provide insight into these issues of perspective and participation, and underlying perceptions of what the field ‘is’, including the object of study and the creation of associated problematics. As we also show, funding streams, often from government sources, play an important role in shaping the semantic constitution of migration studies and directing research towards specific issues, often because of their policy relevance.

## Data and methods

To develop the arguments, we established an original DMRI dataset, which includes institutes with research specifically dedicated to international migration and that were operative for at least one year in the period between 1 January 1945 and 1 January 2020.^2^

This focus on dedicated institutes captures only part of research on human migration because relevant research also takes place in non-dedicated institutes with broader scope. For example, the DMRI dataset includes 117 European institutes, but it has been estimated that over 3,500 organisations in Europe have published research on migration in the period 2004 to 2018—although most of these organisations published only one or a handful of articles (Levy, 2020). We acknowledge that a significant share of migration research, possibly most of it, does not happen at dedicated institutes, but, equally, there can be little doubt that there has been strong growth of the number of centres and institutes as well as networks that bring them together, such as IMISCOE (International Migration Research Network) in Europe that had—by the end of 2022—63 member institutes or centres bringing together more than 1,000 researchers. By focusing on dedicated centres or institutes, we can better understand where and why the topic of migration has become part of an institutional apparatus and are, often, considered worthy of public funding.

We recognise that the boundaries of what counts as international migration research are fluid. It would clearly be beyond the scope of this article to define what topics make up migration studies, but fortunately other projects provide greater taxonomical clarity.^3^ We include in the DMRI dataset all organisations that have established formalised structures explicitly dedicated to research on international migration—either in their name or in the main description of the institute found on the official webpage. We distinguish between academic institutes (educational institutions dedicated to education and research, which grant academic degrees), think tanks (institutes that perform research and advocacy, both non-governmental and semi-autonomous agencies within government or are associated with political parties or businesses), and networks (formal collaborations with their own sources of funding). We use the terms centres or institutes to describe these groupings, although other terms are also used such as ‘group’ or ‘network’. We recognise that this may lead to variations on organisational mission and identity, but are main interest is in the institutionalisation of research, its location and the themes that are addressed.

By including these various kinds of centres or institutes in the DMRI dataset, we are not judging the quality of the research that is undertaken. We chose an inclusive approach, but allow users to customise the DMRI dataset which is available open access. In addition to the DMRI dataset, we also provide interactive visualisations that should facilitate consultation of the data (https://tinyurl.com/2kczaj6y).

We built the DMRI dataset in four steps. First, we searched the Web of Science Core Collection database on SCI-EXPANDED and SSCI with the keyword ‘Migration Studies’. We extracted the 1,061 most cited articles from the search, then tracked the information on the affiliation of the authors of the articles to create the first version of the data.^4^ We only included in the DMRI the institutes that matched the following two criteria: (1) they are explicitly dedicated to the study of migration (i.e., either in their title or in the description of the core activities); (2) they are designed as permanent (i.e., we did not include projects that are designed to be time-limited). We were initially able to include 214 items in the database. As a second step, we published an open access version of the DMRI dataset online and we invited researchers to contact us to expand the DMRI dataset with missing institutes. Through this iteration, we were able to include fifty additional items from thirty-two countries globally. As a third step, we contacted twelve social science researchers from countries that were under-represented despite having a sizeable population: Bangladesh, India, Indonesia, the People's Republic of China, the Russian Federation, Nigeria, Pakistan. We received information on two new items. Finally, we contacted all institutes in the DMRI dataset asking to revise the data we had and to suggest additional centres that were still missing. We thus added sixteen items from nine countries in the Americas, Europe, and Asia. While we relied heavily on crowdsourcing, the entire process was supervised by a central team in charge of ensuring the consistency of the criteria of inclusion/exclusion.^5^

Analytically, we extend the description of research institutes over time and space with bivariate and multivariate analysis at the country level. The Bayesian regression models are equivalent to OLS and use the default uninformative defaults in the R package rstanarm (Goodrich et al., 2020). These priors regularise but do not influence the posterior, drawing on the distribution of the observed values in the data without introducing subjective beliefs. Depending on the model, the outcomes are whether there is a research institute on migration in a country (binary variable), or the number of researchers in these institutes in 2020. As predictors we use the log of GDP per capita and the net migration share of the country in which the centre or institute is located. We present the median of the posterior as coefficients, and as robust measures of uncertainty—equivalent to standard errors—the median absolute deviation (MAD). In the bivariate analysis, we also consider the Human Development Index (HDI) of the country in which the centre or institute is located, an indicator of ‘migration intensity’ which we measure as the absolute value of migration flows and that allows us to identify countries with high net levels of immigration *or* emigration, and an indicator of whether ethnic minorities are included in national legislatures (Ruedin, 2009).

## A trend towards global migration studies?

### Evolution over time

**Fig. 1:**
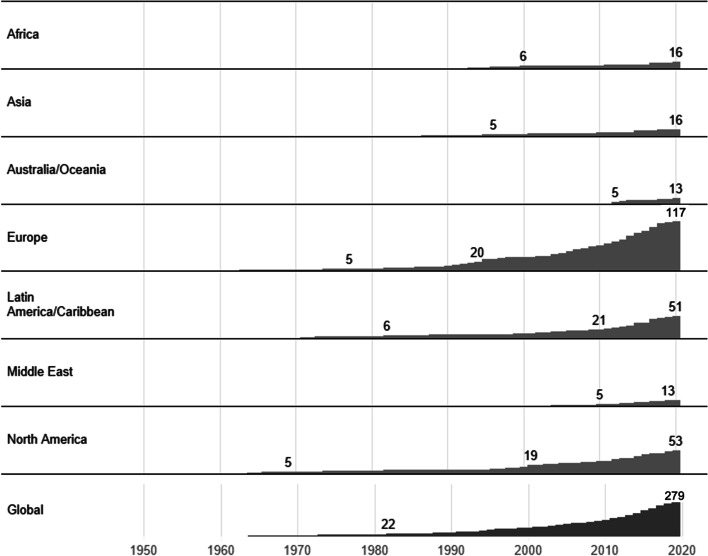
Timeline: number of institutes dedicated to the study of human migration. *Notes*: Own elaboration

Even the oldest institutes dedicated to the study of international migration that were in operation in 2020 have a relatively recent history. Only one was founded before World War II—the *International Union for the Scientific Study of Population* (IUSSP) established in Brussels in 1928—and few others were established shortly after the end of World War II—e. g., the *Peace Research Institute Oslo* (PRIO) established in 1959. However, Fig. 1 shows that it was only from the 1990s that these institutes started to spread globally: In 1995, there were 53 institutes dedicated to the study of international migration spread between the Americas, Asia, and Europe. The most significant acceleration took place in the 2010s, when the number of institutes more than doubled, with an increase from 118 in 2009 to 279 in 2020. Of the total 282 institutes in the database, only three have terminated their operations. On average, between 1990 and 2020, eight new institutes were created every year.

While we cannot identify with the data at hand what events and processes triggered institutional development, the literature provides two plausible suggestions: dedicated funding programmes and international conferences.

First, the establishment of dedicated institutes was encouraged by the growing attention to migration in national funding schemes, such as those that were made available to researchers in the US, in Europe and via research funding from the European Union (EU) (Scholten, 2018). With the emergence of international initiatives and cross-border collaborations, there has also been a diversification of the topics—integration, refugee studies, transnationalism—and a greater number of both researchers and research institutes, most notably (but not only) in Europe (Levy et al., 2020). A Europeanisation of research funding linked specifically to the EU was associated with the idea that migration was a ‘challenge’ closely linked to European integration. The creation of the International Migration, Integration and Social Cohesion in Europe (IMISCOE) network, now known as the International Migration Research Network, was specifically enabled by EU funding. The IMISCOE network organised conferences, workshops and seminars while also offering support to PhD researchers through its graduate network. In turn, this encouraged the creation of new institutes dedicated to the study of migration that joined IMISCOE (Levy, 2020). Other specific funding initiatives such as the UK Research and Innovation Global Challenges Fund have also triggered the creation of new research centres or institutes (Kofman, 2020, p. 2).

A key role was played by international conferences and events that can prompt the creation of institutes dedicated to research on migration. For instance, the Third Joint Conference of the Pakistan-India People’s Forum for Peace and Democracy (1996), a four-day gathering of 400 peace activists in Calcutta, marked the beginning of the *Mahanirban Calcutta Research Group*, now known as the *Calcutta Research Group* (CRG), established by a group of researchers, trade unionists, feminist thinkers and women’s rights campaigners, academics, journalists, and lawyers. It is possible that the recent adoption of the Global Compact for Migration (GMC)—at a United Nations-convened conference notably—had similar generative effects: Between 2018 and 2020, 37 new institutes dedicated to the study of migration have been created, an average of 12 every year. Some of these institutions explicitly refer the GCM in their mission statements. The *African Migration Observatory* in Rabat, for example, was established by Decision 695/2018 of the Assembly of Heads of State and Government of the African Union (AU) to facilitate the implementation of the GMC through data collection, the promotion of continental and international cooperation while also strengthening the contribution of migration to sustainable development.

### Geographical distribution across world regions

**Fig. 2:**
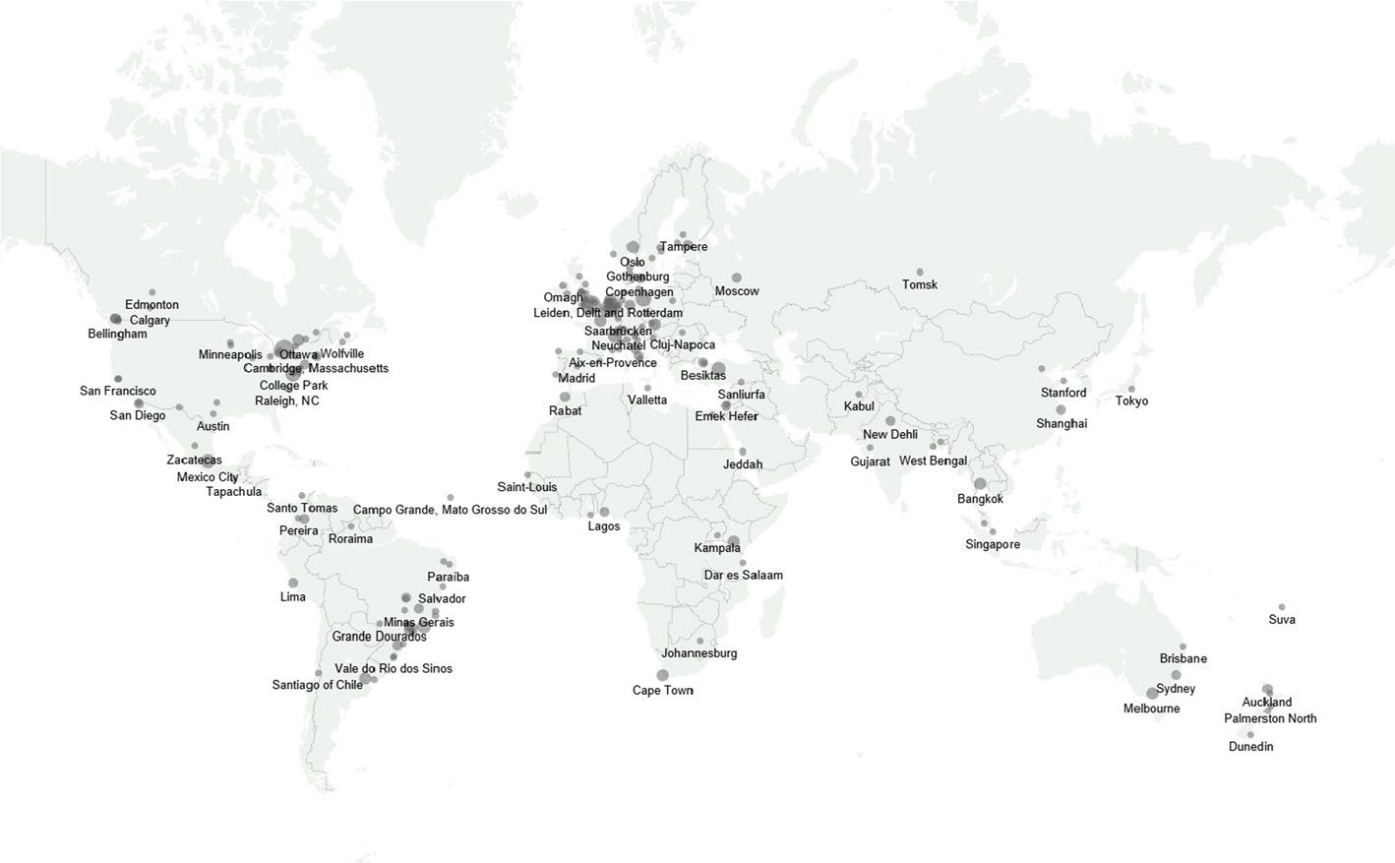
Map of the 279 institutes dedicated to the study of human migration active in 2020. *Notes*: Own elaboration. We place a dot in every city with a migration-research institute active in 2020. The size of the dot is proportional to the number of institutes in the city: the largest dots are in London and Toronto, with eight institutions each

The DMRI makes it very clear that the infrastructure of knowledge production in the field of migration studies is uneven. Figure 2 locates the institutes on a map. It is immediately apparent that most of the institutes are in the Americas (especially in Brazil, 31, the United States, 29, and Canada, 25) and in Europe (especially in the United Kingdom, 22, Germany, 12, the Netherlands, 11, Belgium and Italy, 10). The Americas and Europe combined have 80 per cent of all the institutes (222 out of 282). We have been able to identify comparatively few institutes, or 20 per cent of the total, in Africa, Asia, the Middle East, and Oceania. Remarkably, we identified fewer than 10 institutes in China, Indonesia, and India combined (population of around 3 billion) compared to 25 in Canada (population 37 million). In other words, the existence of migration institutes cannot be explained simply by looking at a country’s size or population.

**Fig. 3:**
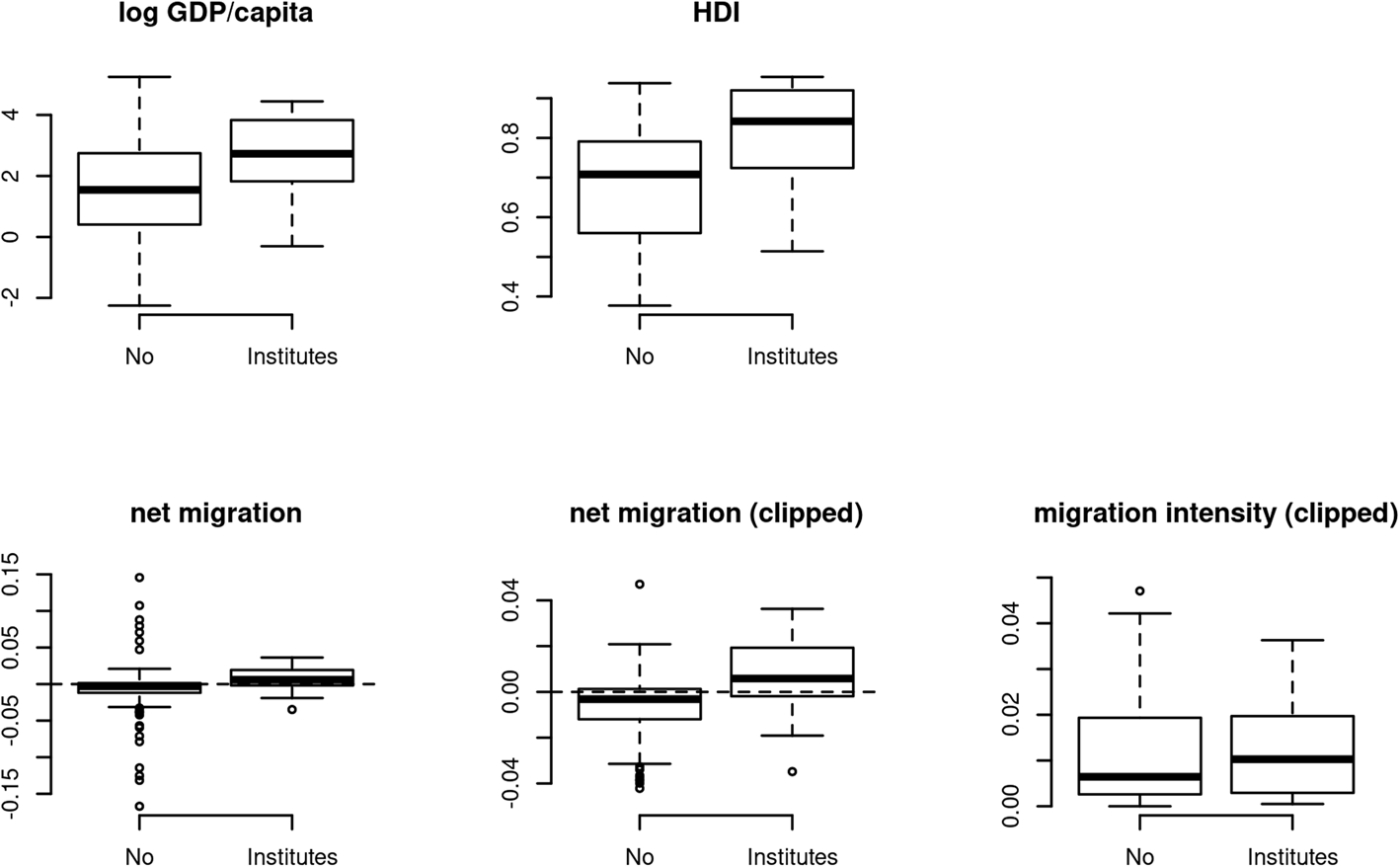
Countries with and without research institutes on migration by GDP, HDI, and migration flows, 2020. *Notes*: Net migration measures is expressed as a share of the population, the clipped graph does not show values above 0.05 and below -0.05, the vertical dashed line indicates zero: countries above this line are net destination countries, countries below this line are net origin countries; migration intensity is the absolute value of the net migration, not showing values above 0.05

It could be assumed that the number of institutes per country is related to the magnitude or scale of the phenomena associated with international migration, but Fig. 3 demonstrates that this is only partially the case and can also be associated with the institutionalisation of higher education and research. While some countries with a high number of immigrants or emigrants have many research institutes—i.e., the Netherlands (11), Switzerland (8), and Mexico (9)—others do not. For example, countries of the Middle East have high numbers of immigrants, but we were able to identify only 13 institutes in the entire area.

Figure 3 demonstrates some correlation between migration flows and the presence of migration institutes. The figure measures whether there is an association between the number of dedicated research institutes in a country and the country’s GDP, HDI, immigration and emigration flows in 2020. The countries with both the highest emigration and immigration flows generally do not have dedicated institutes; by contrast, countries with migration institutes tend to be countries of net immigration. Almost all the inter-quartile range of the boxblot for countries with migration institutes is above the dashed zero line in the figure. A different way to see this is presented in the bottom-right panel of the figure, where we look at the absolute value of migration flows—high values for high levels of emigration *or* immigration. There is no clear difference in this case.^6^ Instead, we can clearly see that migration institutes are concentrated among the richer and more developed countries. For both the log of per capita GDP and for the HDI can we see a clear association with having at least one dedicated institute.

These patterns are also confirmed in the regression analysis presented in Table 1, where we jointly considered GDP and migration flows. The coefficient for the log GDP per capita is positive and noticeably larger than the measure of uncertainty in brackets for all the models, indicating a clear pattern: richer countries tend to have more migration institutes (M1), even after accounting for net migration. They also tend to have a larger number of institutes per country (M2), a larger total number of researchers in these institutes (M3), and institutes with more employees (M4).

**Table 1:** Having at least one migration institute, number of institutes and researchers, regression models, 2020

	M1	M2	M3	M4
log GDP per capita	0.12 (0.02)	0.95 (0.23)	63.3 (31.1)	4.2 (2.3)
Net migration	1.24 (0.02)	8.89 (9.96)	4766 (2930)	16.8 (202.5)
(Intercept)	0.09 (0.05)	− 0.19 (0.50)	− 32.8 (82.1)	14.4 (5.8)

Outcome = country has at least one migration institute (M1, binary indicator), number of migration institutes in countries (M2, range from 0 to 31), total number of researchers in migration institutes in country (M3, range from 2 to 1420), median number of researchers in migration institutes in country (M4, range from 2 to 69). Shown is the median of the posterior as coefficients, with the median absolute deviation (MAD) as a measure of uncertainty in brackets. Net migration as a share of the population. Minority representation is a binary indicator, measuring whether members of ethnic minority groups are represented in national legislatures. N = 183 observations in M1, N = 53 for M2 to M4 (only countries with institutes). Priors are default Normal(0, 2.5) scaled to the data

We note, however, that some upper middle-income countries have created a research funding system similar to those in high income countries, leading to a high number of institutes. Brazil, for example, has 31 research institutes dedicated to migration, a higher number than the US, which has 29. Argentina (9) and Turkey (8) also have relatively high numbers of research institutes. Yet, in these countries the infrastructural institutionalisation has not produced a distinct impact on migration studies, largely because of the dominance of English as lingua franca of migration studies from the start of the twenty-first century (Pisarevskaya et al., 2019, p. 467).

We provide further evidence of such uneven distribution between high-income and low-income countries in the annex: Appendix 1 lists the institutions with more than 100 publications in nine migration-related journals listed on Web of Science between 1975 and 2021. All 36 institutions in the list are in six high-income countries and in five of these countries English is the mother-tongue: Australia, Canada, the Netherlands, Singapore, the UK, the US.

Looking at net migration, we can see a positive association in most cases, even after accounting for differences in GDP. Larger net immigration (as opposed to emigration) is associated with a greater likelihood of having a migration institute at all (M1), with a larger number of migration institutes in the country (M2), and with a larger number of researchers in these institutes overall (M3). By contrast, for the association between net migration and the median size of the institute, the uncertainty of the estimate is much larger than the effect size, and we refrain from interpreting this coefficient.

### From the infrastructural to the semantic organisation of the field

There are multiple hurdles for researchers coming from countries that do not have a strong infrastructure in place, including fewer possibilities to be hired and limits to access and publication in international scientific outlets (Khan, 2020). By contrast, due to their infrastructural dominance, high income ‘destination’ countries have been able to collect migration data more systematically and develop key theories in the field of migration studies. In other words, high income ‘destination’ countries have had enhanced capacities to shape the semantic orientations of the field, or the main of subjects of interest, concepts, narratives, and priorities.

This can be explained also by looking at the origins of this infrastructure which, as we suggested, largely lie in dedicated funding streams and conferences. Traditionally, such factors have facilitated the emergence of specific topics that reflected European and North American perspectives. For example, International Metropolis funded originally by Citizenship and Immigration Canada and the Social Sciences and Humanities Research Council (Canada) in 1996 set up four institutes initially, whose research explicitly aimed to serve Canadian policy makers and service providers. As a result, research has given significantly more prominence to certain topics, such as integration, smuggling and trafficking, remote control; and has paid less attention to other topics, such as work recruitment agencies, educational mobility, and multinational migrations (Achiume, 2019; Triandafyllidou, 2022; Collins, 2022).

It is not inevitable, however, that institutes in high income countries only pursue research relevant to the interests of those specific sites. Indeed, institutes may well vary in the type of research and their relationship with partners abroad. Some researchers in high income countries have advanced migration theory often based on work conducted in or with partners in low-income countries. Researchers from PRIO, for example, have conducted research in the Global South for decades. Instances of ideas originating from such engagement are evident in theorisation of the aspiration/ability model of migration (Carling & Schewel, 2018).

There are growing numbers of institutes from high-income countries conducting research in the South and engaging in collaborations with partners in the South. This is also due to changes in the requirements of funding bodies and the growing number of international development research bodies (Canada, European Commission, Denmark, Sweden, UK) that support research conducted either together with or by organisations in the Global South. Recent European Research Consortium (ERC) and Horizon Programmes funded by the European Commission, for example, can provide resources to establish collaborations in countries of the Global South. These funding streams enable the development of partnerships, joint events, shared research programmes and data collection projects. They also tend to be defined by the policy priorities within the EU institutions and have an applied policy focus. Reflecting EU priorities, this has led to an increased focus on the development of partnerships by EU-based researchers with researchers outside the EU that also reflect the policy preoccupations of the EU with ‘migration governance’, broadly understood. In practical terms, this means that the semantic field of research in the European context—where there has been steep growth in infrastructural development—is also structured by funding from the EU that is driven by policy concerns and the preoccupations that inform them. To be selected for funding a project must demonstrate that it meets the requirements of the call and that it has a highly developed impact strategy to engage with relevant stakeholders, including EU policymakers. This does not mean that the researchers involved with these projects must only work within the frame of reference determined by the policy framework because many of these projects have developed insights and evidence that are highly critical of current policy.

In turn, this has then raised concerns about the relationship between migration research and policy and whether research evidence actually has much influence on policy. The underlying rationale for EU funding this type of applied, policy focused research does tend to rest on an assumption about a particular relationship between evidence and policy that, as has been extensively documented may well not hold (Boswell, 2009). This can be because policy priorities are already established and difficult to change or because evidence is cherry picked to justify existing choices. In short, the semantic constitution of the field also raises questions not only about the field of ‘migration studies’ itself, but also about its relationship to political authority in the multi-level European system.

These can also be seen as issues associated with the ‘migration studies’ field itself as an example of what has been referred to as ‘post normal science’ when ‘facts [are] uncertain, values in dispute, stakes high and decisions urgent’ (Funtowicz and Ravetz, 1994). While the European and EU system is distinct from those in other regions, we have shown that its institutional and semantic characteristics give the EU system a prominent place in the wider, global dynamics of research on migration and, because of the funding arrangements, also demonstrate the relevance of links to funding authorities and their priorities.

## Conclusion

The DMRI shows that countries in Europe and the Americas host about two-thirds of the institutes dedicated to the study of migration. The geographical concentration of institutes in these regions is largely driven by economic development (GDP) and net immigration. We find that equivalent patterns of net emigration are not associated with having migration institutes. We interpret the geographical distribution as influenced by political concerns with specific problems related to the regulation of how people move, and the rights movers should be entitled to. This specific infrastructure leads to a distinct understanding of what constitutes a ‘subject of interest’ in migration studies, driven by specific concerns and ideas (Smith, 2021). The concentration of institutes in specific places shapes the research agenda along the assumptions, concepts, interests, priorities, and topics that are of more immediate concern for those living in high-income ‘destination’ countries.

We suggest that this geographical imbalance is key in explaining why epistemic communities in migration studies are formed and reproduced mainly in those high-income ‘destination’ countries. Because migration scholars from low-income countries have limited access to funding, publishing, and training, it is more difficult to turn their knowledge into institutionalised academic knowledge in the first place. As a consequence, adopting ‘inclusive and transformative approaches, language and categories of doing migration research’ (African Academy for Migration Research, 2021) may not suffice to address existing biases. We show that uneven knowledge production is not only an issue of language or inclusive journal policies but also a structural problem at an earlier stage: Comparatively (too) few resources are invested in the creation of institutionalised academic knowledge in low-income countries. This is not an issue for migration studies alone, as efforts to decolonise research in the social sciences and humanities underscore (Smith, 2021; Collins 2022). Indeed, in our view further work should be dedicated to understanding the growth and concentration of higher education and university research in a variety of different disciplines, as well as the uneven diffusion of research institutes dedicated to other transnational topics such as climate, gender, and trade.

Geographical inequalities pose pressing challenges for migration studies. It is particularly striking that we could identify remarkably few dedicated institutes in Africa, Asia, the Middle East, and the Gulf Countries. In part, that is a reflection of general inequalities: Even where African, Asian, Middle East, and Gulf authors are writing, they are often hired by American or European institutes (Chan, 2020). Mapping the geographical patterns on dedicated institutes provides a quantitative description of Global North–South inequalities and contributes to explaining why the research and policy agenda in the field of migration is largely produced by scholars who are overwhelmingly based in high-income ‘destination’ countries.

While the development of a truly global network of migration scholars still seems some way off, there are some actions that could be taken to avoid that scholars in low-income countries remain at the margins of the discipline. This includes, for example, setting up partnerships, collaborative research initiatives, joint organisation of trainings, summer schools, and workshops that would allow for infrastructural reinforcement but also for a more inclusive dialogue on the semantic constitution of the migration studies field.

## Data Availability

The data that support the findings of this study is available online at Piccoli, 2023. Interactive visualisations that facilitate consultation of the data are available at: https://tinyurl.com/2kczaj6y.

## Appendix 1: Institutions with more than 100 publications in migration-related journals, 1975–2021

**Table:**

Institution	Number of publications	Country
University of London	796	United Kingdom
University of California	727	United States
University of Oxford	419	United Kingdom
University of Amsterdam	274	Netherlands
University of Sussex	270	United Kingdom
University of California Los Angeles	248	United States
City University of New York	222	United States
London School of Economics and Politics	216	United Kingdom
University of Texas	184	United States
University of Bristol	171	United Kingdom
State University New York	167	United States
University College London	165	United Kingdom
University of Toronto	164	Canada
University of Manchester	160	United Kingdom
Goldsmiths University London	156	United Kingdom
University of Warwick	143	United Kingdom
University of Wisconsin	143	United States
State University of Florida	139	United States
California State University	138	United States
National University of Singapore	136	Singapore
Utrecht University	133	Netherlands
University of Leeds	131	United Kingdom
University of Southampton	129	United Kingdom
York University of Canada	128	Canada
University of Texas	124	United States
Pennsylvania Commonwealth University	123	United States
Harvard University	120	United States
University of California Davis	120	United States
Princeton University	119	United States
Radbound University Nijmegen	114	Netherlands
Australian National University	112	Australia
Columbia University	112	United States
University of Surrey	112	United Kingdom
University of Illinois	106	United States
University of California Berkeley	102	United States
New York University	101	United States

Web of Science search, selection of articles that are currently indexed in the SSCI and published in nine migration-related journals: *Asian and Pacific Migration Journal* (APMJ), *Ethnic and Racial Studies* (ERS), *European Journal of Migration and Law* (EJML), *International Migration* (IM), *International Migration Review* (IMR), *Journal of Ethnic and Migration Studies* (JEMS), *Journal of Refugee Studies* (JRS), *Mobilities*, *Population Space and Place* (PSP).

## Abbreviations

AU: African Union
CRG: Calcutta Research Group
DMRI: Directory of Migration Research Institutions
ERC: European Research Consortium
EU: European Union
GCM: Global compact for migration
GDP: Gross domestic product
HDI: Human development index
IMISCOE: International migration research network
IUSSP: International Union for the Scientific Study of Population
MAD: Median absolute deviation
PRIO: Peace Research Institute Oslo
SCI-EXPANDED: Science citation index expanded
SSCI: Social sciences citation index

## Footnotes

1 For example, more than hundred researchers, all working on EU funded research projects, wrote to high level EU officials to protest that policy decisions ran directly counter to the findings of their research https://admigov.eu/upload/Call_EU_funded_researchers.pdf.

2 The information in the dataset has been checked to the best of our knowledge. However, in some cases our interpretation may be incorrect or there may be some institutes that we have overlooked. We invite readers to let us know if they find errors, inconsistencies, or missing institutes. If we learn about incomplete or wrong information, we will correct them. We plan to periodically publish an updated version of the dataset, also expanding the temporal coverage beyond 2020.

3 For further information, see the CrossMigration project which itself brought together many IMISCOE centres and institutes from across Europe to conduct a state-of-the-art overview of the migration studies field, including the creation of a Migration Research Hub: https://migrationresearch.com/.

4 We acknowledge that there is a bias built into using highly cited papers as a starting point for the analysis given that this will effectively place emphasis on Western institutions and minimise the appearance of institutions where researchers operate in languages other than English. Our focus on articles published in English is, however, a reflection of the field, which increasingly came to adopt “English as the lingua-franca for academic research on migration in a rather organic manner” (Pisarevskaya et al., 2019, p. 477). We also believe that our triangulation ensures that this bias is greatly abated.

5 The dataset includes variables that we do not explore here, such as the gender of the director of a given institute in 2020. While roughly half of the directors are female, in Africa and Oceania the ratio men-women is clearly imbalanced (88% male directors in Africa, 17%, male directors in Oceania). We also include in the dataset the textual description that appears on the institution’s homepage for future analyses.

6 We also considered the share of asylum seekers and notice that the mean share of asylum seekers in countries with institutes and in countries without is almost identical. Moreover, we looked at minority representation as a predictor to capture cultural or historical differences—countries where there is more attention to minority population may also be more attuned to the particularities of migrant populations. Of countries with minority representation 34% have migration institutes, of those without minority representation, 26% have migration institutes.
